# Pollen development in *Annona cherimola *Mill. (Annonaceae). Implications for the evolution of aggregated pollen

**DOI:** 10.1186/1471-2229-9-129

**Published:** 2009-10-29

**Authors:** Jorge Lora, Pilar S Testillano, Maria C Risueño, Jose I Hormaza, Maria Herrero

**Affiliations:** 1 Estación Experimental "La Mayora", CSIC, 29760 Algarrobo-Costa, Málaga, Spain; 2 Centro de Investigaciones Biológicas, CSIC, Ramiro de Maeztu 9, 28040, Madrid, Spain; 3 Dep. Pomología, Estación Experimental "Aula Dei", CSIC, Apdo. 202/50080 Zaragoza, Spain

## Abstract

**Background:**

In most flowering plants, pollen is dispersed as monads. However, aggregated pollen shedding in groups of four or more pollen grains has arisen independently several times during angiosperm evolution. The reasons behind this phenomenon are largely unknown. In this study, we followed pollen development in *Annona cherimola*, a basal angiosperm species that releases pollen in groups of four, to investigate how pollen ontogeny may explain the rise and establishment of this character. We followed pollen development using immunolocalization and cytochemical characterization of changes occurring from anther differentiation to pollen dehiscence.

**Results:**

Our results show that, following tetrad formation, a delay in the dissolution of the pollen mother cell wall and tapetal chamber is a key event that holds the four microspores together in a confined tapetal chamber, allowing them to rotate and then bind through the aperture sites through small pectin bridges, followed by joint sporopollenin deposition.

**Conclusion:**

Pollen grouping could be the result of relatively minor ontogenetic changes beneficial for pollen transfer or/and protection from desiccation. Comparison of these events with those recorded in the recent pollen developmental mutants in Arabidopsis indicates that several failures during tetrad dissolution may convert to a common recurring phenotype that has evolved independently several times, whenever this grouping conferred advantages for pollen transfer.

## Background

Pollen development is a well characterized and highly conserved process in flowering plants [1-3]. Typically, following anther differentiation, a sporogenous tissue develops within the anthers producing microsporocytes or pollen mother cells. Prior to meiosis, pollen mother cells become isolated by a wall with the deposition of a callose layer. Each pollen mother cell, as the result of the two meiotic divisions, generates four haploid cells forming a tetrad and, for a short time, these four sibling microspores are held together in a persistent pollen mother cell wall that is surrounded by callose. The tapetum then produces an enzyme cocktail that dissolves the pollen mother cell wall and the microspores are shed free and become independent [2]. The unicellular microspores go through an asymmetric mitotic division (pollen mitosis I) to produce a pollen grain with two cells, a larger vegetative cell that hosts a smaller generative cell; the latter will divide once more to produce two sperm cells (Pollen mitosis II). Pollen mitosis II can take place before or after pollen release and, depending on when it occurs, the pollen will be bicellular or tricellular at the time of anther dehiscence. Throughout the manuscript we will use the term "pollen tetrads" for mature pollen to avoid confusion with the tetrads of early developmental stages ("microspore tetrads").

Angiosperms pollen is most commonly released as single pollen grains or monads [4] which represent the basic angiosperm pollen-unit. Dehiscence of aggregated pollen (mostly in groups of four) is considered a recent apomorphic characteristic [5,6] that has arisen independently several times during evolution primarily in animal-pollinated taxa although, in some cases, monads may have evolved secondarily from groups of four grains [6]. Pollen release as tetrads has been reported in some or all members of 55 different angiosperm families and also in some pteridophytes [7]. Blackmore and Crane (1988) [8] put forward that the maintenance of pollen tetrads could be the result of relatively minor ontogenetic changes and, consequently, this could be an excellent example of convergence in situations where the release of pollen as tetrads is an effective reproductive strategy. Interestingly, the dissemination of pollen as tetrads has also been reported in the *quartet *mutants of *Arabidopsis *[9,10].

Annonaceae, included in the order Magnoliales, is the largest family within the basal angiosperm Magnoliid clade [11,12]. Due to its phylogenetic position among the basal angiosperms, the family has been the object of considerable interest from a taxonomic and phylogenetic point of view [13-15] and a number of studies have focused on pollen morphology [16-20]. Although most genera of the Annonaceae produce solitary pollen at maturity, in several species of the family pollen is released aggregated in groups of four or in polyads [17]. Recent studies on the mechanism of pollen cohesion in this family have been performed in species of the genera *Pseuduvaria *[21], *Annona *and *Cymbopetalum *[22,23]. Pollen cohesion in these species is generally acalymmate (four pollen grains are grouped only by partial fusion) with simple cohesion [21]. But these studies show differences in cohesion mechanisms; thus, while pollen grains in *Pseuduvaria *are connected by wall bridges (crosswall cohesion), involving both the exine and the intine, in *A. glabra, A. montana *and *Cymbopetalum *cohesion is achieved through a mass of callose-cellulose. Evolutionary transitions in flowering plant reproduction are proving to have a clear potential in plant evolutionary biology [24], and the need for more detailed ontogenetic studies in the family has been put forward [22]. Indeed the fact of being the largest family among basal angiosperms, together with the puzzling connection mechanisms so far described in the different species examined, provide an excellent opportunity to investigate the ontogeny of pollen development and its evolutionary implications.

In this work, pollen development is characterized in *A. cherimola*, one of the species in the Annonaceae where pollen is shed aggregated in groups of four, paying special attention to the events close to pollen formation and retention of the individual pollen grains together, observed by immunolocalization of different wall components. Results are discussed in relation to the shedding of pollen in groups of four in other species and how this event may have occurred and settled during evolution.

## Results

The mature *A. cherimola *flower is a syncarpous gynoecium with a conic shape composed of about 100 fused carpels surrounded at its base by several rows of anthers with up to 200 stamens, encircled by two whorls of three petals. The flower cycle from opening to anther dehiscence lasts two days: the flower opens on the morning of the first day in the female stage and remains in this stage until the afternoon of the following day when the flower enters the male stage. Anther dehiscence occurs concomitantly in all stamens of a flower and, as the anthers dehisce, they detach from the flower and fall over the open petals.

Flower buds of *A. cherimola *develop in the leaf axes following leaf expansion; the basal nodes are differentiated in the year preceding anthesis. The uppermost distal buds differentiate in synchrony with shoot growth [25]. Flower bud growth begins 39 days prior to anthesis. Anther differentiation proceeded centripetally, with the most developmentally advanced anthers placed in the outermost rows and the different stages of anther and pollen development present within the same flower. This fact was helpful for establishing successive stages of anther development. The anther becomes septate with pollen mother cells positioned between rows of interstitial tapetum similar to the anthers described in a sister species, *Annona squamosa *[26].

To determine if pollen development followed a standard pattern and whether pollen tetrads at anther dehiscence corresponds with the cytological and morphological features of mature pollen, anther and pollen development were examined from microsporogenesis to maturity. Special attention was given to the events responsible for pollen cohesion. Microgametogenesis and tapetum degeneration were also examined sequentially.

### Microsporogenesis

**Figure 1:**
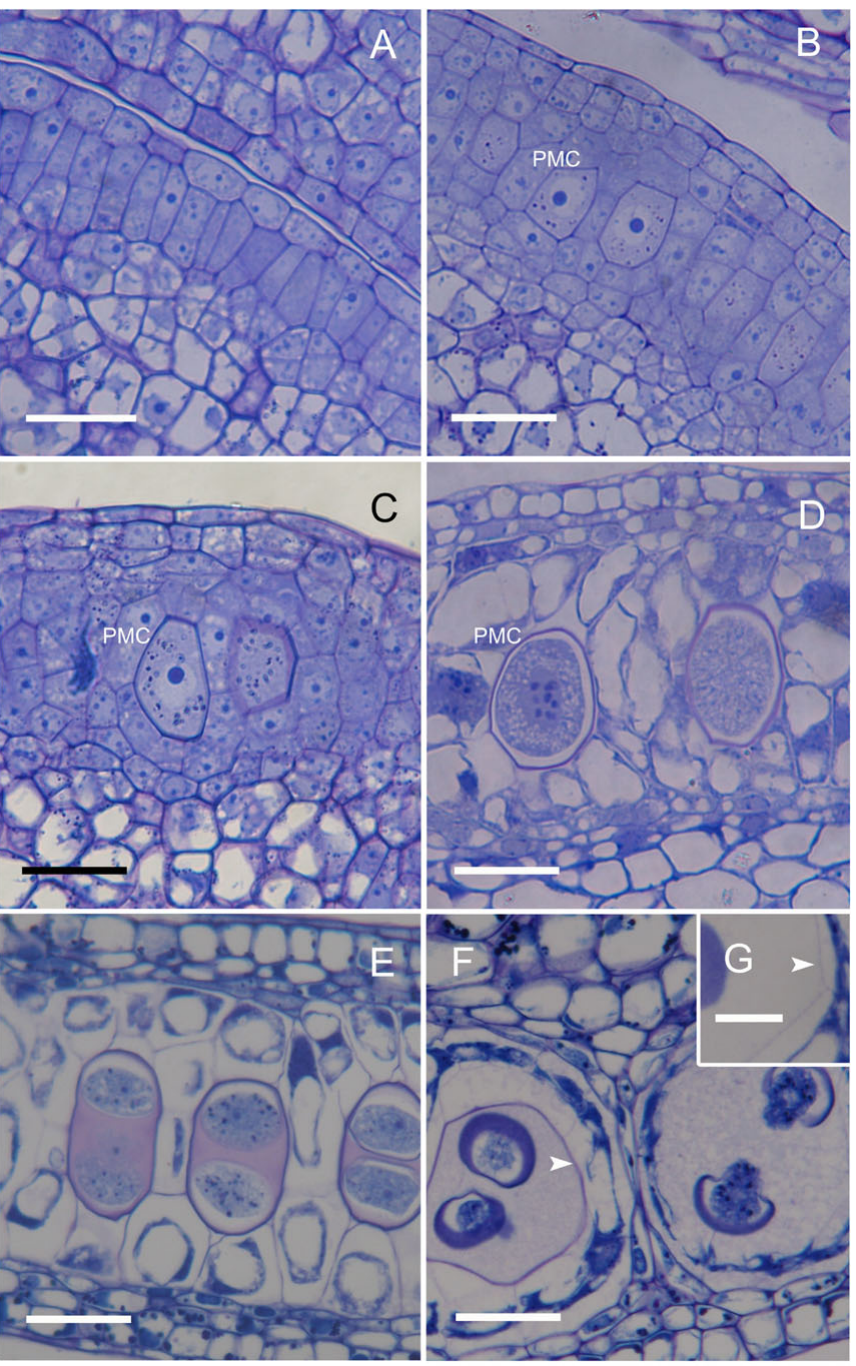
**Microsporogenesis of *Annona cherimola***. (A) Uniseriate row of arquesporial cells. (B) Septal and pollen mother cells (PMC), showing a conspicuous wall, alternate in the sporogenous tissue. (C) PMC increase in size and starch grains are visible. (D) Starch grains vanish, a translucent layer appears in the PMC wall, and PMC starts meiosis. The tapetum vacuolates and the tapetal chambers are apparent. (E) Following meiosis, starch grains accumulate again in the young microspores, which are surrounded by callose. (F) The young microspores, with an incipient exine, appear to float and turn within the still remaining PMC wall (arrow) that holds the four microspores together. (G) Detail of PMC wall (arrow). Longitudinal sections of the anthers stained with PAS and Toluidine blue. Bar = 20 μm.

Initial hypodermal archesporial cells were apparent 24 days before anthesis (Figure 1A). From them, anther septa initials and pollen mother cells (PMC) developed in 9 cm long flower buds 19 days before anthesis (Figure 1B). Each anther contained a uniseriate row of pollen mother cells with a conspicuous common wall. The PMC began to accumulate starch grains (Figure 1C) and increased in size (Figure 1C, 1D). Starch grains vanished concomitantly with the beginning of meiosis, some 15 days before anthesis, as a translucent cell wall layer was apparent surrounding the PMC (Figure 1D). Meiosis proceeded rapidly and was followed by a new accumulation of starch grains in the young microspores (Figure 1E) 14 days before anthesis. The microspore tetrads remained together in isolated tapetal chambers surrounded by the PMC wall that stained positively with periodic acid-Shiff's reagent (PAS) for carbohydrates (Figure 1F, 1G).

**Figure 2:**
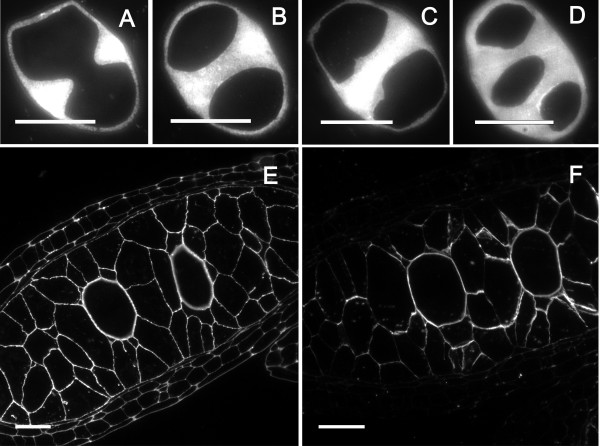
**Callose and pectins during microsporogenesis in *Annona cherimola***. (A-D) Anticallose in dyad/tetrad phases. (A) A furrow of callose developed inwards, forming a wall between the dyad cells. (B) Dyad phase, showing (B) an incipient, and (C) a well developed callose wall. (D) Tetrad microspore showing in the section plane three of the microspores separated by callose walls. (E) PMC and other anther tissue walls showing methyl-esterified pectins. (F) PMC wall also shows unesterified pectins. Specific cell components were localized using antibodies against callose (A, B, C, D), methyl-esterified pectin (JIM7) (E), and unesterified pectin (JIM5) (F). A-D Bar = 10 μm. E-F: Bar = 20 μm.

Immunocytochemical essays revealed the localization of various cell wall components (Figure 2). Callose surrounded the PMC wall and, following meiosis I, an additional furrow of callose developed inwards (Figure 2A) forming a callose positive band between the dyad cells (Figure 2B, 2C). Successive cytokinesis followed (Figure 2C), resulting in a tetrad (2D), each separated by callose. Dyad and tetrad stages coexist in the flower as centripetal maturation progresses. The PMC wall also reacted positively to JIM7 (Figure 2E) and JIM5 (Figure 2F) staining, indicating the presence of methyl-esterified and unesterified pectins respectively. However, while the walls of the anther somatic and tapetal cells also reacted positively to the JIM7 for methyl-esterified pectins (Figure 2E), they showed only a faint staining for the presence of unesterified pectins (Figure 2F).

### Pollen cohesion

**Figure 3:**
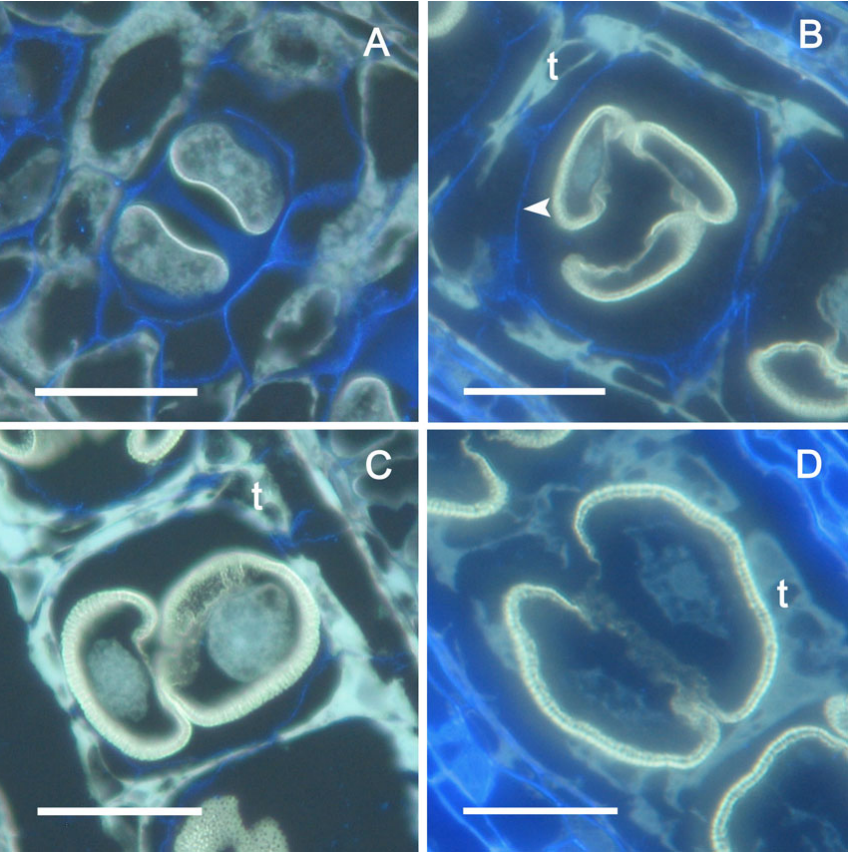
**Pollen development within the tapetal chamber in *Annona cherimola***. (A) Two young microspores in a tetrad which still keeps the pollen mother cell wall. Aperture sites are located towards the outside facing the pollen mother cell wall. (B) Pollen is shed free, within the PMC wall, in the tapetal chamber. Within this confined space the young microspores turn (C) with their aperture sites facing now each other as the PMC cellulosic wall is digested. (D) The pollen grains regroup sticking through the aperture sites, and enlarge as the tapetum degenerates. Longitudinal anther 2 μm resin sections stained with calcofluor and auramine. Bar = 20 μm.

Following tetrad formation, callose disappeared but the microspores remained within the PMC calcofluor-positive cellulosic wall (Figure 3A). At this developmental stage, an interesting event was detected: the microspores within each tapetal chamber, which initially had their pollen aperture sites facing outward towards the PMC wall (Figure 3A), appeared to float and rotate within their individual chambers (Figure 3B). This movement was not random, but the microspores turned 180° until the pollen aperture sites faced each other (Figure 3B). The remaining PMC cellulosic wall, which persisted for some time, together with the confined space provided by the tapetal chamber, appear to contribute towards keeping the microspore tetrad together. Subsequently the PMC cellulosic wall disappeared completely (Figure 3C) and the tapetum degenerated as the microspores increased in size. They remained in their new orientation attached by their apparently sticky aperture sites that now faced each other (Figure 3D).

**Figure 4:**
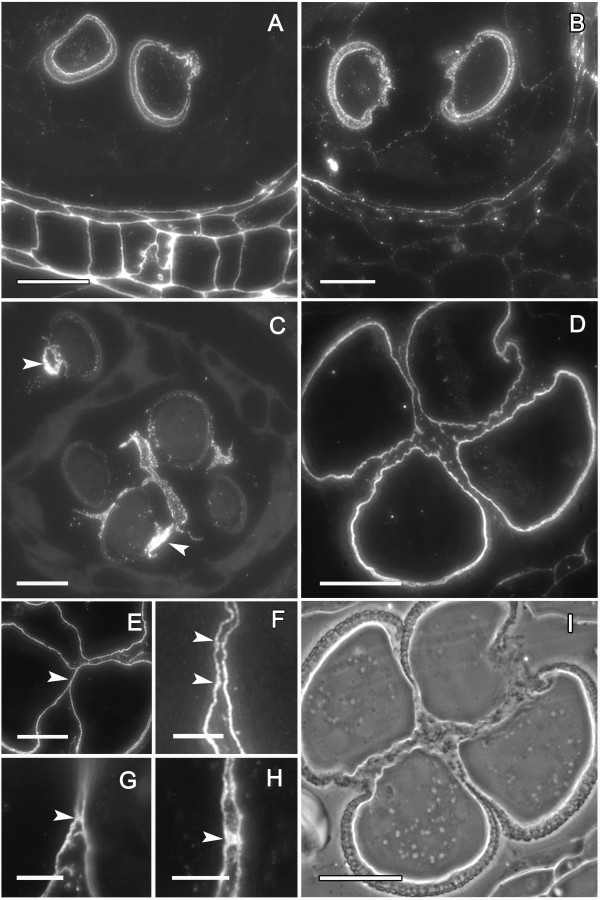
**Establishment of pollen cohesion in *Annona cherimola***. (A) Microspore walls show methyl-esterified pectins, and also (B) unesterified pectins. (C) As callose is digested, remnants of callose (white arrow) are observed layering the pollen aperture sites. (D-F) Microspores show crosswall cohesion bridges showing the presence of unesterified pectins. (G-H) Details of crosswall cohesion bridges, showing the presence of methyl-esterified pectins. (I) Phase contrast of a mature pollen grain showing internal cohesion and a joint sporopollenin layering. Specific cell components were localized using antibodies against: methyl-esterified pectin (JIM7) (A, G-H), unesterified pectin (JIM5) (B, D, E, F) Callose (C). A-E, I: Bar = 10 μm. F-H: Bar = 3 μm.

At this stage, both the cell walls of the somatic cells of the anther and the inner wall of microspores (intine) reacted similarly for methyl-esterified pectins (Figure 4A), while unesterified pectins were present just in the microspore intine (Figure 4B). The exine showed a low unspecific autofluorescence but in a different fluorescence wavelength (yellowish color) than the fluorescent marker of the antibodies, AlexaFluor 488, which emitted green fluorescence. As a consequence, exine autofluorescence was clearly differentiated from the immunofluorescence signals. Anti-callose immunofluorescence revealed remnants of callose at the pollen aperture sites where the thick external layer of the microspore wall, the exine, was extremely thin or absent. These callose remnants were apparent in all microspores at this stage (Figure 4C). The four microspores showed crosswall cohesion bridges that stained with antibodies against unesterified and methyl-esterified pectins in the microspore wall (Figure 4D-H). Following this inter-intine cohesion, additional deposition of sporopollenin with a joint layering of the four microspores further strengthened this connection (Figure 4I).

### Microgametogenesis

**Figure 5:**
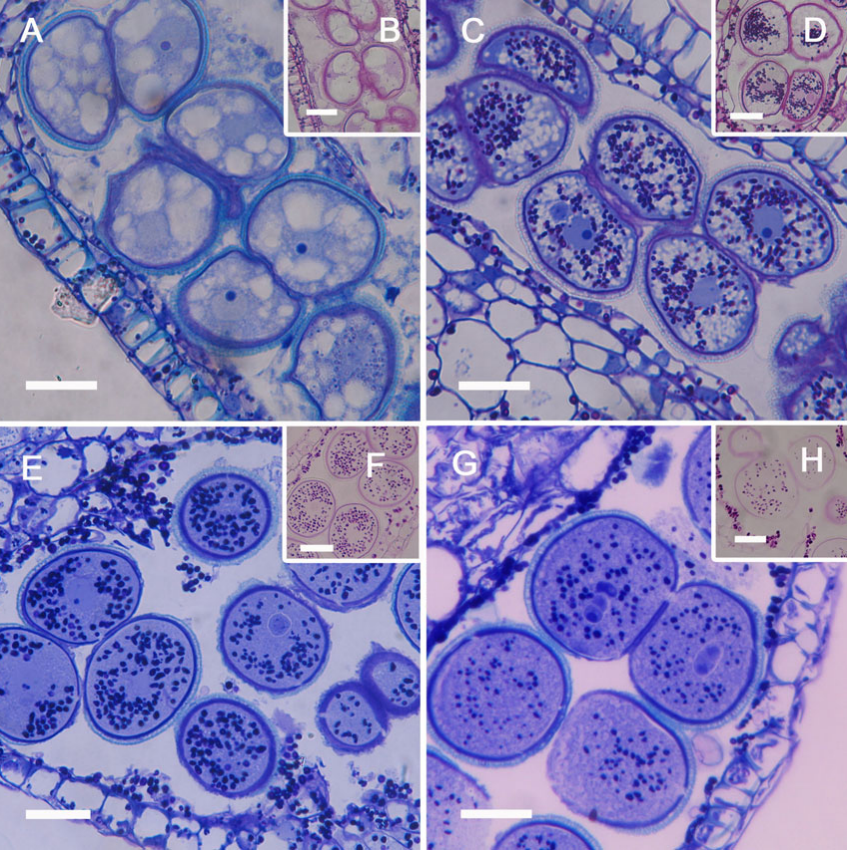
**Microgametogenesis in *Annona cherimola***. (A) Microspores increase in size as vacuoles appear in the cytoplasm, (B) microspores at this stage do not have starch grains. (C) Microspores following mitosis I; (D) as vacuoles decrease in size, starch grains are stored. (E) Young pollen grains without vacuoles (F), which accumulated starch grains. (G) Close to the time of anther dehiscence, the second mitosis occurs, the tapetum is completely degenerated and (H) starch is digested. Longitudinal sections of anthers stained with PAS and Toluidine blue (A, C, E, G), and with PAS (B, D, F, H) to show starch grains. Bar = 20 μm.

As the microspores increased in size, their cytoplasm became vacuolated (Figure 5A) and starch grains were absent (Figure 5B). During this vacuolization, nuclear migration preceded the first mitosis to form bicellular pollen grains. Following the first mitosis, 4-6 days before anthesis, the vacuoles decreased in size (Figure 5C) and starch was again stored (Figure 5D). Young pollen grains had no vacuoles (Figure 5E) and numerous starch grains were present (Figure 5F). The second mitotic division producing the first tricellular pollen grains started some four hours prior to anther dehiscence (Figure 5G) and one day prior to anther dehiscence starch grains began to hydrolyze (Figure 5H). Mitotic divisions were not synchronized within a pollen tetrad and single pollen grains with different numbers of nuclei could be observed in the same tetrad resulting in the coexistence of bicellular and tricellular pollen upon anther dehiscence.

**Figure 6:**
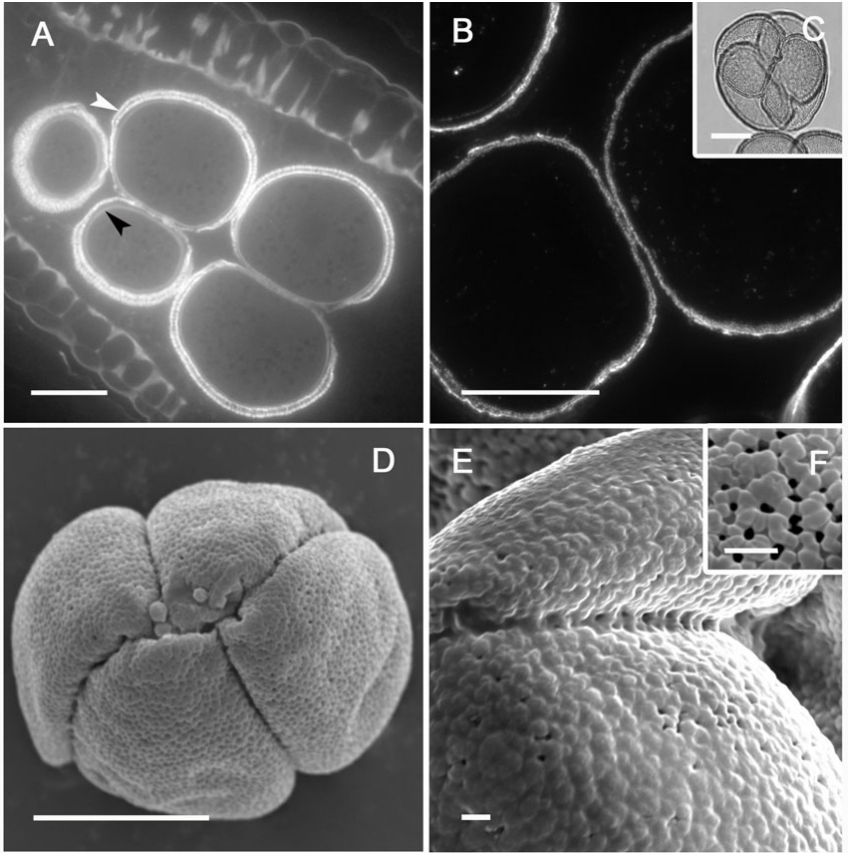
**Mature pollen of *Annona cherimola***. (A) Intine (black arrow) is similar all around the pollen grain, but exine (white arrow) is thinner in the pollen aperture site. Longitudinal section stained with a 3:1 mixture of Auramine and Calcofluor. (B) Sibling pollen grains have a faint cohesion that showed with JIM 5 antibody the presence of unesterified pectins. (C) Mature pollen tetrad following acetolysis. (D, E, F) Mature pollen observed with scanning electron microscopy (SEM). (D) Mature pollen grains with a globose shape and a radiosymmetric disposition. (E) Exine cohesion helps keeping sibling pollen grains together. (F) Pollen exine shows a tectate perforate appearance. A, B, D: Bar = 20 μm; C: Bar = 10 μm; E, F: Bar = 2 μm.

In mature pollen, while intine thickness was similar around the pollen grain, the exine was thinner or absent at the pollen aperture sites where contact points between sibling pollen grains were established (Figure 6A). At these areas unesterified and methyl-esterified pectin bridges were maintained throughout pollen development although these connections seemed to be less strong in mature pollen (Figure 6B). However, mature pollen tetrads resisted separation during acetolysis, showing the permanence of joint sporopollenin (Figure 6C).

Scanning electron micrographs revealed that mature pollen had a radiosymetric globose shape, was inaperturated, tectate perforate, and with a diameter of 40 μm (Figure 6D, 6E, 6F). Mature pollen was shed in groups of four sibling pollen grains that stick together having an exine cohesion, clearly visible with high magnification scanning electron microscopy images (Figure 6E).

### Tapetum degeneration

**Figure 7:**
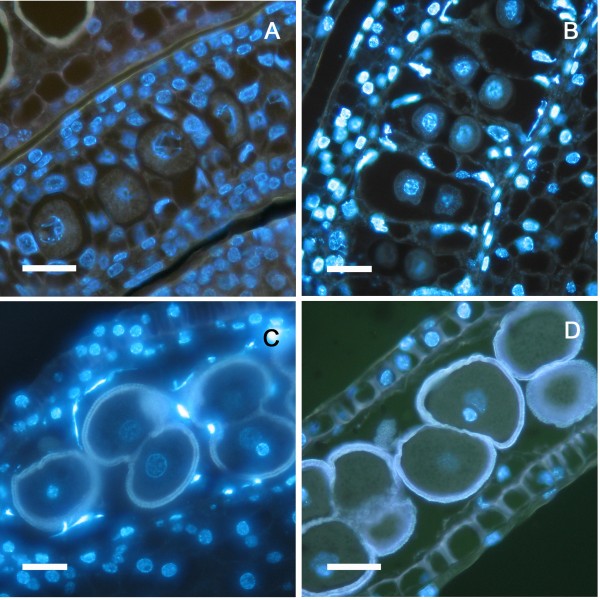
**Tapetum degeneration in *Annona cherimola***. (A) Pollen mother cells in Prophase I and an active tapetum. (B) Dyad phase in enlarged tapetal chambers. (C) Anther, 4 days before anthesis, showing bicellular pollen and degenerated tapetum, with nuclei displaying elongated shapes and chromatin condensation. (D) Tapetum has disappeared in anthers of flowers at the female stage showing mature pollen. Longitudinal 5 μm resin sections stained with DAPI. Bar = 20 μm.

*A. cherimola *has a secretory tapetum with tapetal-type septa similar to those described in other species of the genus *Annona *such as *A. squamosa *[26] and *A. glabra *[27]. Prior to meiosis, septal initials formed tapetal chambers that host the PMC (Figure 7A). After meiosis, the tapetum showed a vacuolization and a progressive degeneration as the tapetal chamber enlarged (Figure 7B). The nuclei of the tapetal cells displayed elongated and lobular shapes together with a extremely high chromatin condensation, revealed by an intense 4',6-diamidino-2-phenylindole (DAPI) fluorescence (Figure 7C), typical features of programmed cell death [28], which have also been found in the tapetal nuclei of other species [29]. At the same time, tapetal cells released their cellular contents that coated the pollen grains to form the pollenkit. At anther dehiscence the tapetum was completely degenerated and had disappeared (Figure 7D).

## Discussion

### Pollen development

Pollen in *A. cherimola *is shed in groups of four, originating from the same meiotic division and, hence, the same tetrad. Pollen development, however, continues beyond tetrad formation and, although held together, pollen grains are fully mature upon anther dehiscence. Meiosis cytokinesis occurred through the formation of ingrowths of callose that are also found in genera of some primitive angiosperms [30,31] including species of the Magnoliid clade as *Magnolia tripetala *[32] and *Degeneria vitiensis *[33] in the Magnoliales, *Laurelia novae-zelandiae *[34] and *Liriodendron tulipifera *[35] in the Laurales or *Asarum *in the Piperales [30] as well as in monocots as *Sisyrinchium *[36].

Starch accumulated prior to meiosis and the first pollen mitosis and vanished with the onset of these two divisions; this also occurred 6 days before anther dehiscence, preceding the shedding of starchless pollen. The accumulation of starch in PMC has also been reported in other primitive angiosperms, such as *Anaxagorea brevipes *[37] or *Austrobaileya maculata *[38], and in other evolutionarily more recent angiosperms [39,40]. Vacuolization also follows a conserved pattern [41,42]. The cytoplasm enlarges through a first vacuolization and, later on, following the first pollen mitosis, small vacuoles appear as starch builds up.

In most species a dehydration process takes place prior to pollen shedding and starch is hydrolyzed to form sucrose that protects pollen against desiccation [43]. Starchless pollen is the most common pollen type in the angiosperms [44], being more frequent in bicellular than in tricellular pollen [45,46]. In *A. cherimola*, pollen is shed in a highly hydrated stage [47] and this lack of dehydration may explain why the second mitotic division continues in free pollen after pollen shedding, producing a mixed population of bi and tricellular pollen [48]. However, both types of pollen are starchless at anther dehiscence.

### Pollen cohesion

Several reasons could account for the release of pollen in groups of four. In *Arabidopsis*, failure of different enzymes during the dissolution of the pectic layer that surrounds the PMC wall has been reported in two *quartet *mutants [10,49]. In our work the immunolocalization of esterified and non-esterified pectins showed that, although they were clearly present in the PMC wall, the pectins disappeared following tetrad formation. A closer examination of the photographs reveals that the PMC wall, which stains for cellulose, remained beyond the tetrad stage. Interestingly *quartet *mutants of *Arabidopsis *also show a defect in the degradation of the PMC wall [10]. Cellulase has also been shown to be involved in the breakdown process of the PMC wall [50] and a delay in its action could lead to this phenomenon. However, this failure does not seem permanent, since 25 days later this wall is completely dissolved. Thus, the permanence of the PMC wall appears as a key factor contributing to pollen grouping as pollen tetrads in *A. cherimola*, similar to the observations in *Arabidopsis *mutants. A mixture of enzymes is require to break down the complex PMC wall [2], and a failure of one or more of these enzymes could result in a similar final result.

Different events that take place during the retention of this wall may explain pollen adherence once this wall disappears. The confining of pollen within the tapetal chamber keeping the young microspores in close proximity may contribute to this wall maintenance. Surprisingly, the young microspores are apparently separated from their sibling cells allowing some free movement indicated by the strange 180° rotation of the pollen aperture sites. Thus, those aperture sites that originally looked outwards rotate inward to face each other. A similar rotation has been reported previously in other Annonaceae [*A. glabra *and *A. montana *[22] and *Cymbopetalum *[23], and also in species of the Poaceae [51]. This distal-proximal microspore polarity transition in development contrasts with the evolutionary shift from a proximal to a distal aperture that has been long regarded as one of the major evolutionary innovations in seed plants [52]. Proximal apertures predominate in the spores of mosses, lycophytes and ferns while distal apertures are more common in extant seed plants including gymnosperms, cycads and early-divergent angiosperms [52]. In fact, species in the Annonaceae with monad pollen are reported to have distal apertures [see [19] for review]. However, a complete study of 25 Annonaceae genera with species that release aggregated pollen showed proximal apertures [16] and, consequently, the distal-proximal transition observed in pollen development of *A. cherimola *and other Annonaceae [22,23] could represent a widespread situation in this basal family.

Another reason proposed for this permanent binding of pollen in groups of four could be a failure in the synthesis of the callose layer during microspore separation in the tetrad [8]. However, the results shown in this work in *A. cherimola *indicate that callose is layered following the standard pattern and vanishes later, after meiosis is completed, similar to the way it occurs in *Arabidopsis quartet *mutants, in which callose dissolution proceeds normally [53]. However, the use of antibodies against callose showed that callose remains for a while in the area where pollen apertures will form hampering the layering of sporopollenin. Callose remnants in this area have also been reported in other Annonaceae and it has been suggested that these remnants pull the pollen grains to undergo the 180° turning [22,23]. In the formation of the pollen wall, callose dissolution occurs concomitantly with the layering of the exine [54] and the formation of the pollen aperture is related to endoplasmic reticulum blocking the deposition of primexine [3]. The callose remnant at the pollen aperture sites has not been investigated in detail in other species and, given the high conservation of pollen ontogeny in angiosperms, this is a topic worthy of a detailed study. Interestingly, in an *Arabidopsis *mutant lacking the gene responsible for callose synthesis, pollen develops unusual pore structures [55].

Further binding at the aperture sites could follow this initial adhesion process through the observed joint deposition of pollenkit that has also been reported in other species [4]. Thus, two key processes could contribute to holding together the four pollen grains in *A. cherimola*, the confined space that permits the delay in the dissolution of the PMC wall and the tapetal chamber and pollen rotation that allows the adhesion of the sticky proximal faces by the formation of small pectin bridges. Later, the join deposition of sporopollenin would further strengthen this initial binding.

### Biological significance of the pollen dispersal unit

A failure or delay in the dissolution of the PMC wall and tapetal chamber appears to be a critical step, resulting in the continued proximity of the four microspores produced by meiosis of a single PMC. However, this phenotype could also result from failure in the different enzymes that dissolve the PMC wall. The distribution of this character, together with the information provided by *Arabidopsis *mutants, shows that this has occurred independently several times during evolution, suggesting that it must provide some evolutionary advantages [8].

The adaptive advantages derived from aggregated pollen have been reviewed recently [6]. The release of aggregated pollen in insect pollinated species could increase pollination efficiency, since more pollen grains could be transferred in a single pollinator visit and, in this sense, a correlation between pollen tetrads and polyads with a high number of ovules per flower has been shown in a survey of the Annonaceae [17]. The release of aggregated pollen is more advantageous in situations where pollinators are infrequent [6] and in situations of short pollen viability and pollen transport periods. A short pollen viability period has been reported in *A. cherimola*, [47,56] and a short pollen transport episode is common in several Annonaceae [57] and in other beetle pollinated species of early divergent angiosperm lineages [58].

An additional possible benefit of aggregated pollen is protection against desiccation and entry of pathogens through the thin walls of the pollen aperture sites. Pollen grouped in dyads, tetrads or polyads show a strong proximal reduction of the exine in Annonaceae [59]. *A. cherimola *pollen is inaperturate and germinates in the proximal face, showing a large area of unprotected intine [47,60]. More evolutionarily recent species present a *colpus *that, in dehydrated pollen, is just a narrow slit protected by loose pollenkit. Only upon hydration, when the pollen faces a wet surface on the stigma, this slit swells developing a wider colpus through which the pollen tube protrudes [61]. Inaperturate pollen does not have this protection from desiccation and the development of inward facing intines may play a role in protecting pollen against desiccation.

## Conclusion

The results obtained in this work support the hypothesis that aggregated pollen could be the result of relatively minor ontogenetic changes beneficial for pollen transfer or/and protection from pollen desiccation. Comparison of the events reported here with those recorded in recent pollen development mutants in *Arabidopsis *suggests that a simple event along development, the delay in the dissolution of the pollen mother cell wall and tapetal chamber, results in conspicuous morphological changes that lead to the release of pollen in tetrads. A variety of different mutations within the enzymes required to breakdown this wall, may contribute to this common morphology. These changes have occurred and recur in nature and, due to their adaptive advantages for pollen transfer, have been selected during evolution several independent times, representing an example of convergent evolution.

## Methods

### Plant material

The research was performed on adult *A. cherimola*, cv. Campas trees of located in a field cultivar collection at the EE la Mayora CSIC, Málaga, Spain. To study the relationship between flower bud length and developmental stages, tagged flower buds were measured sequentially on the trees. Buds were measured twice a week for 8 weeks from leaf unfolding, when the buds were visible but buried under the leaf petiole until anthesis. *A. cherimola*, as other members of the Annonaceae, presents protogynous dichogamy [62]. The flower opens in the female stage and remains in this stage until the following day in the afternoon when at a precise time, around 6 pm. under our environmental conditions, it changes to the male stage: the anthers dehisce, the petals open more widely and the stigmas shrivel [48].

### Light microscope preparations

To follow pollen development, anthers were collected from flower buds of a range of stages, with petals 6, 9, 12, 16, 22, 24 and 30 mm long. Anthers were also collected from flowers one day prior to anthesis and at the female (F) and male (M) stages of mature flowers. The anthers from three flowers of each stage were fixed in glutaraldehyde at 2.5% in 0.03 M phosphate buffer [63], dehydrated in an ethanol series, embedded in Technovit 7100 (Kulzer & Co, Wehrheim, Germany), and sectioned at 2 μm.

Sections were stained with periodic acid-Schiff's reagent (PAS) for insoluble carbohydrates and with PAS/Toluidine Blue for general histological observations [64]. Sections were also stained for cutine and exine with 0.01% auramine in 0.05 M phosphate buffer [65] and for cellulose with 0.007% calcofluor in water [66]. Intine and exine were observed with a 3:1 mixture of 0.01% auramine in water and 0.007% calcofluor in water.

To observe nuclei during pollen development, anthers collected from flowers at the same developmental stages ranging from 9 mm long to anthesis were also fixed in 3:1 (V1/V2) ethanol-acetic acid, embedded as described above, sectioned at 5 μm and stained with a solution of 0.25 mg/ml of 4',6-diamidino-2-phenylindole (DAPI) and 0.1 mg/ml *p*-phenylenediamine (added to reduce fading) in 0.05 M Tris (pH 7.2) for 1 hr at room temperature in a light-free environment [67]. Preparations were observed under an epifluorescent Leica DM LB2 microscope with 340-380 and LP 425 filters for auramine, calcofluor, and DAPI.

For the study of pollen morphology and pollen size, dehisced anthers were sieved through a 0.26 mm mesh sieve and the pollen was placed in glacial acetic acid for acetolysis. Pollen grains were transferred to a mixture of 9:1 acetic anhydride:concentrated sulphuric acid at 65°C for 10 minutes, then washed with glacial acetic acid and washed again three times with water following a modification of the method by Erdtman (1960) [68].

### Scanning electron microscopy

Pollen for scanning electron microscopy (SEM) was fresh dried with silica gel and directly attached to SEM stubs using adhesive carbon tabs and observed with a JSM-840 scanning electron microscope (JEOL) operated at 10 kV.

### Immunocytochemistry

Immunocytochemistry was performed on Technovit 8100 (Kulzer & Co, Wehrheim, Germany) embedded semithin sections and revealed by fluorochromes, as described previously [69,70]. Anthers from three flowers per developmental stage with petals 6, 9, 12, 16, 22, 24 and 30 mm long and at anthesis were fixed in 4% paraformaldehyde in phosphate buffered saline (PBS) at pH 7.3 overnight at 4°C, dehydrated in an acetone series, embedded in Technovit 8100 (Kulzer), polymerized at 4°C and sectioned at 2 μm. Sections were placed in a drop of water on a slide covered with 3-Aminopropyltrietoxy-silane 2% and dried at room temperature.

Different antibodies were used to localize specific cell components: an anti-RNA mouse monoclonal antibody, D44 [71,72], for total RNA detection; JIM5 and JIM7 rat monoclonal antibodies (Professor Keith Roberts, John Innes Centre, Norwich, UK) which respectively recognize low and high-methyl-esterified pectins [73] for localization of pectins; and an anti-callose mouse monoclonal antibody (Biosupplies, Parkville, Australia) for callose.

Sections were incubated with PBS for 5 minutes and later with 5% bovine serum albumin (BSA) in PBS for 5 minutes. Then, different sections were incubated for one hour with the primary antibodies: JIM5, JIM7, and anti-RNA undiluted and anti-callose diluted 1/20 in PBS. After three washes in PBS, the sections were incubated for 45 minutes in the dark with the corresponding secondary antibodies (anti-rat, for JIM5 and JIM7, and anti-mouse, for anti-RNA and anti-callose) conjugated with Alexa 488 fluorochrome (Molecular Probes, Eugene, Oregon, USA) and diluted 1/25 in PBS. After three washes in PBS and water, the sections were mounted in Mowiol 4-88 (Polysciences), examined with a Zeiss Axioplan epifluorescent microscope, and photographed with a CCD Digital Leica DFC 350 FX camera.

## Authors' contributions

JL performed most of the experimental analyses, PST had an active contribution to the immunocytochemistry assays, MCR designed and discussed the immunocytochemistry essays, JIH participated in the design of the experiments, MH coordinated the study. All authors contributed to the draft and read and approved the final manuscript.
